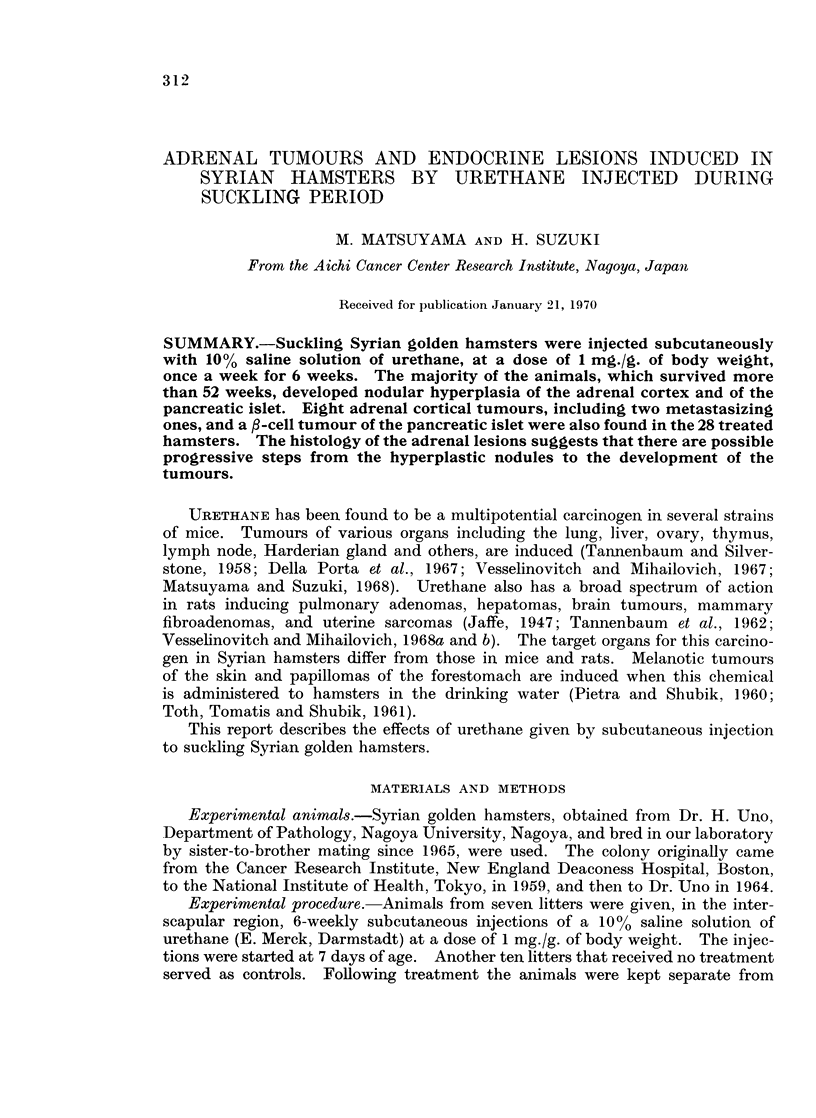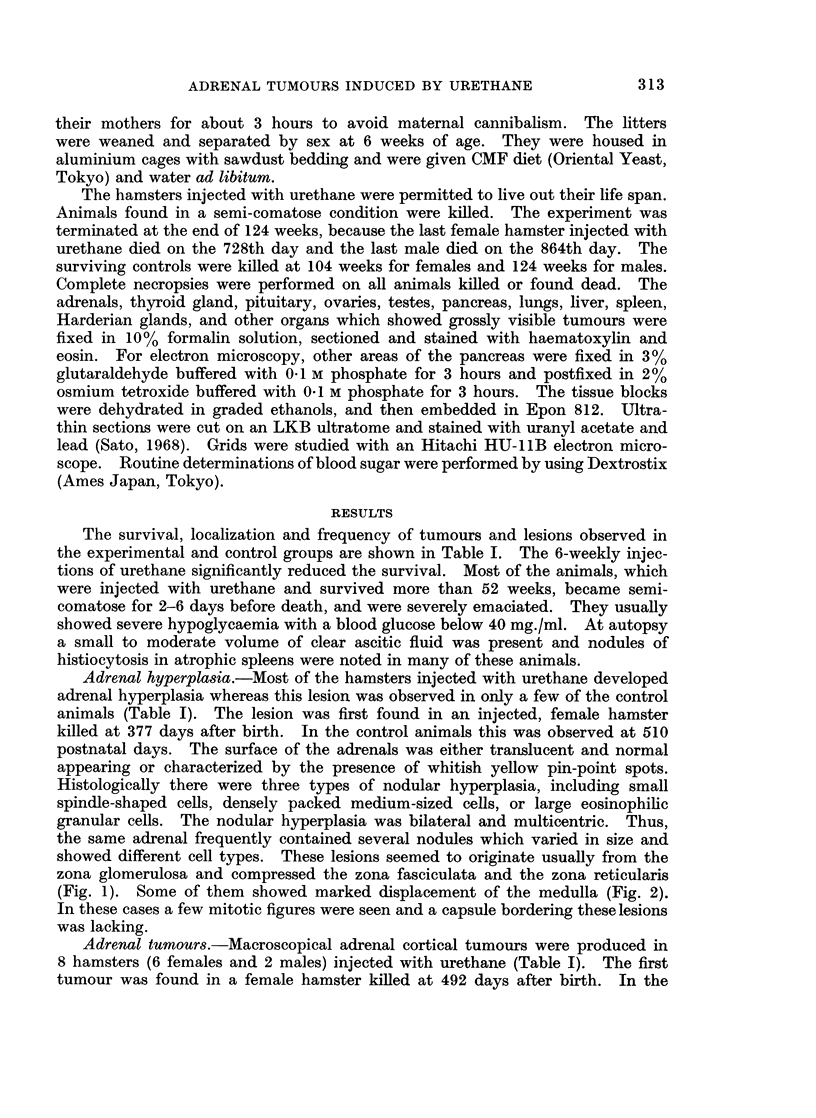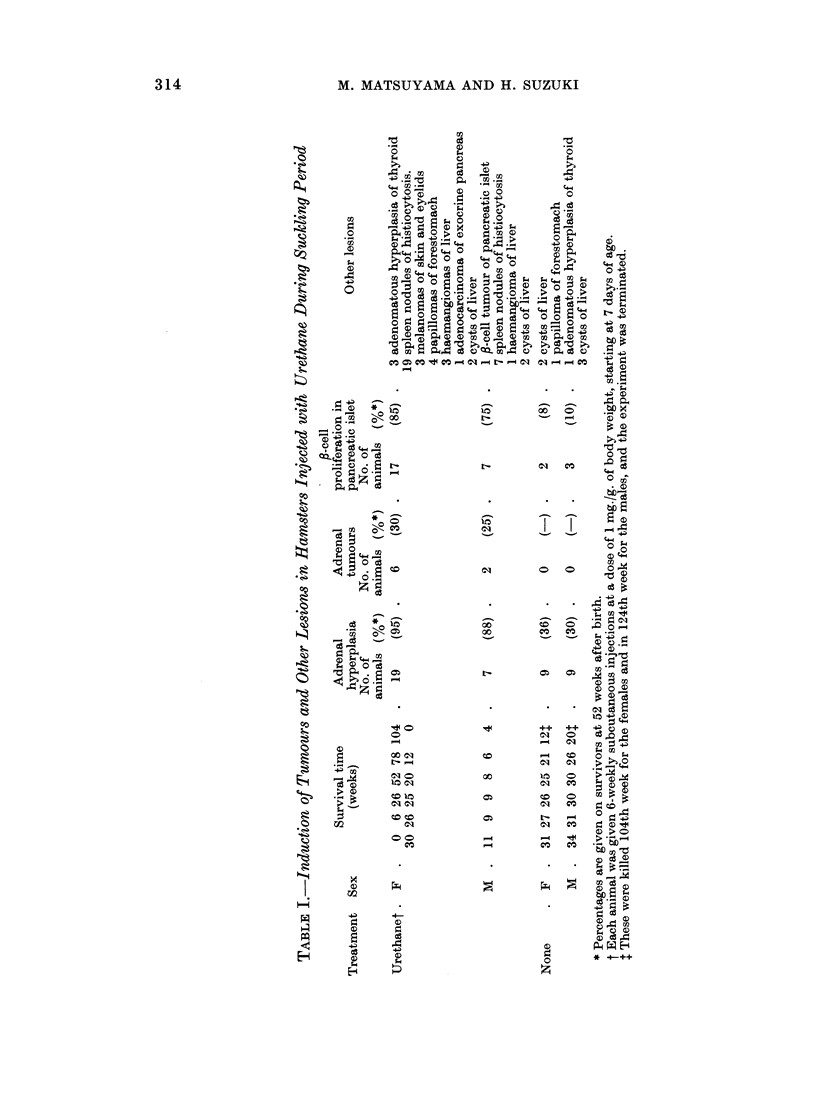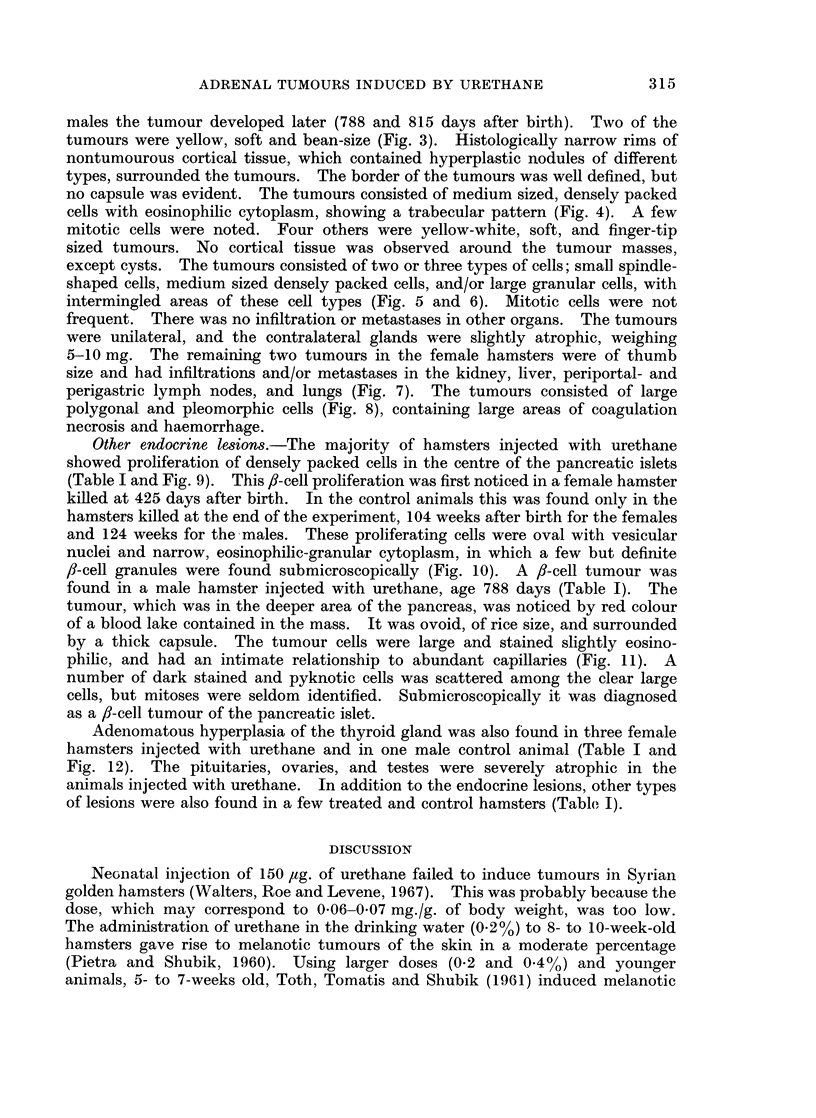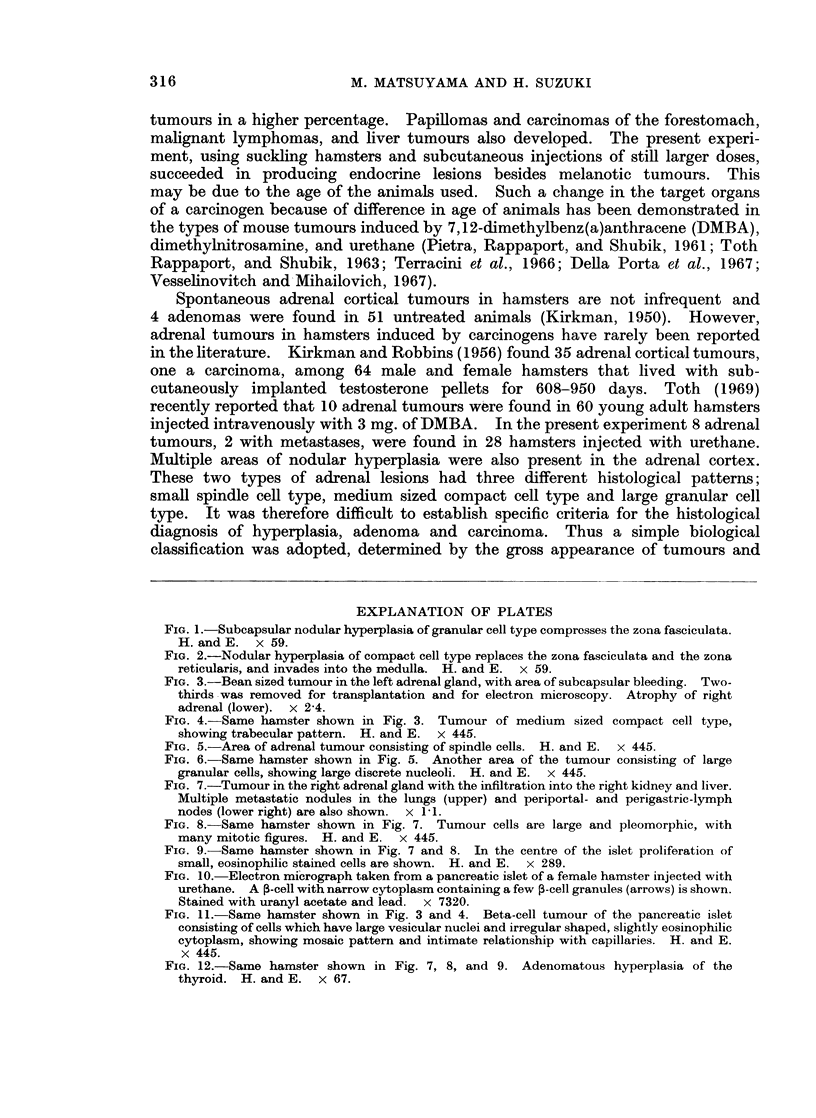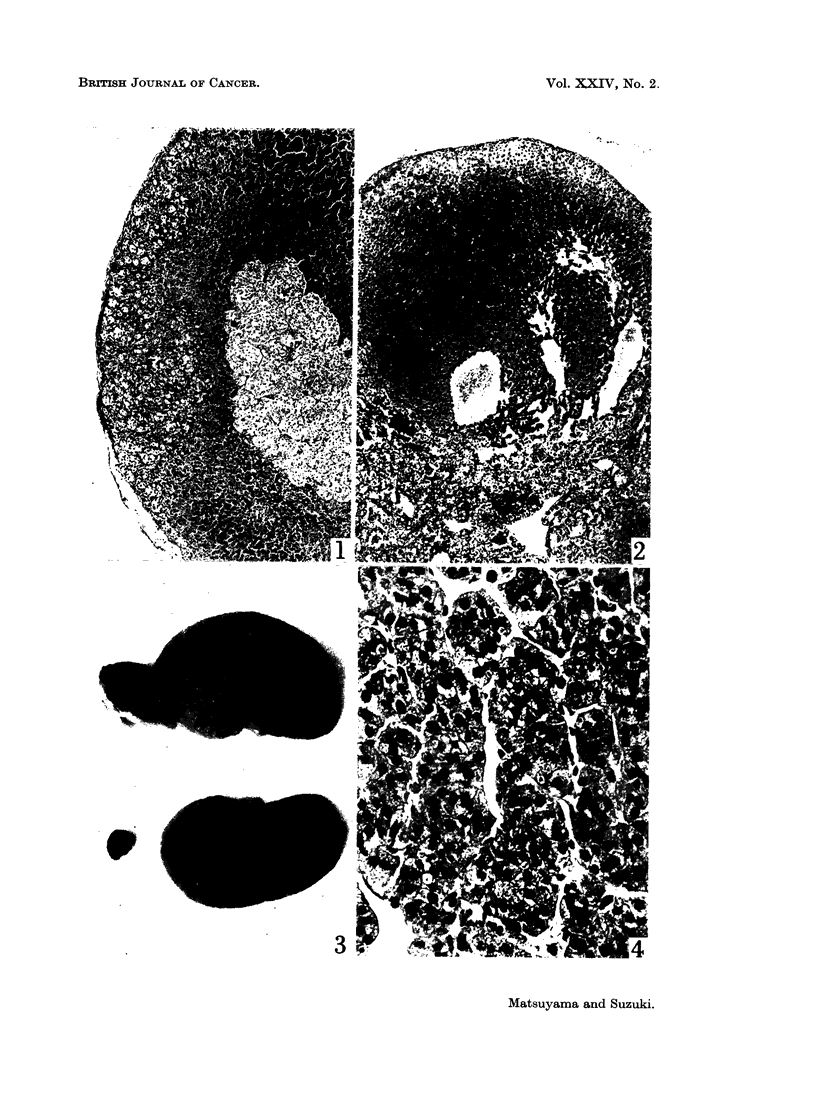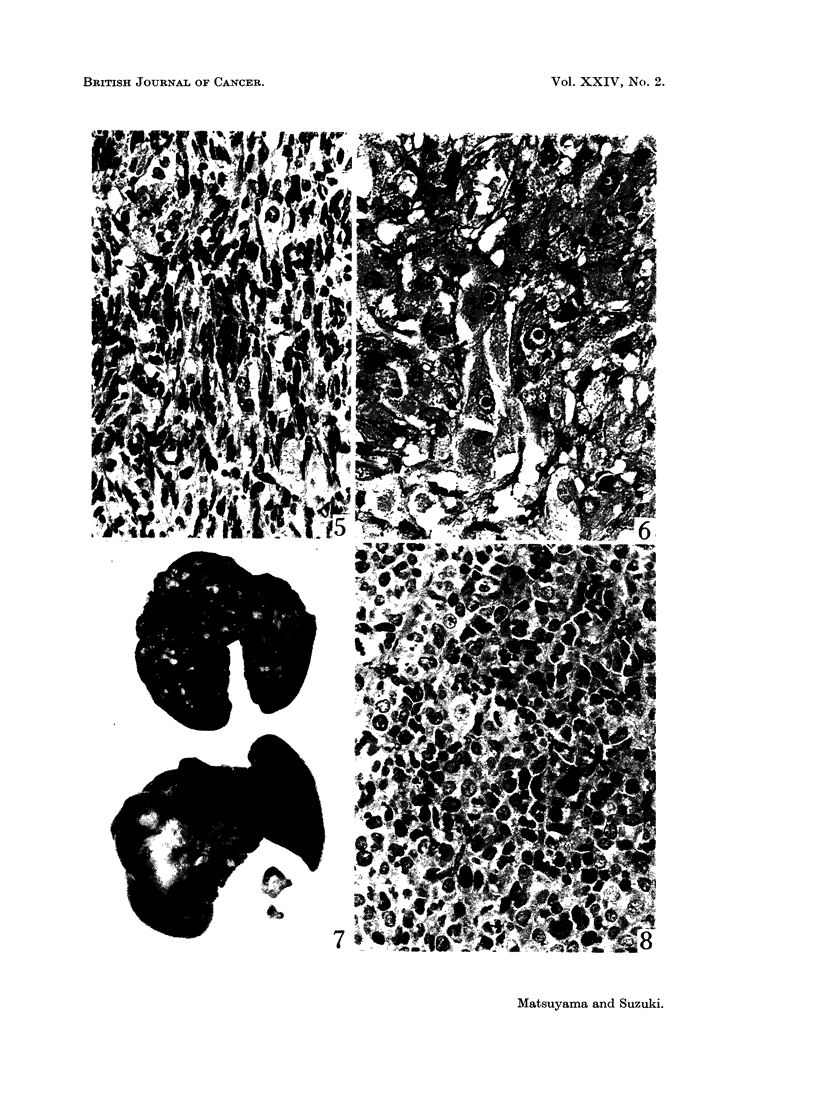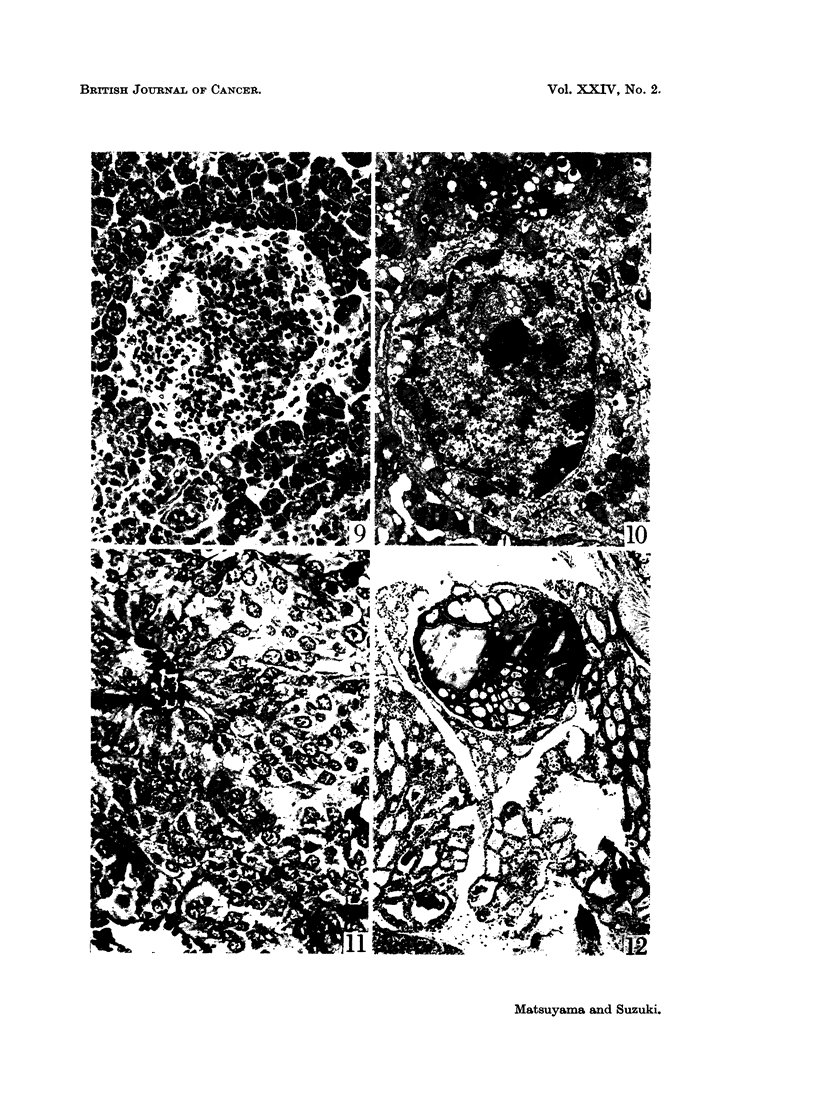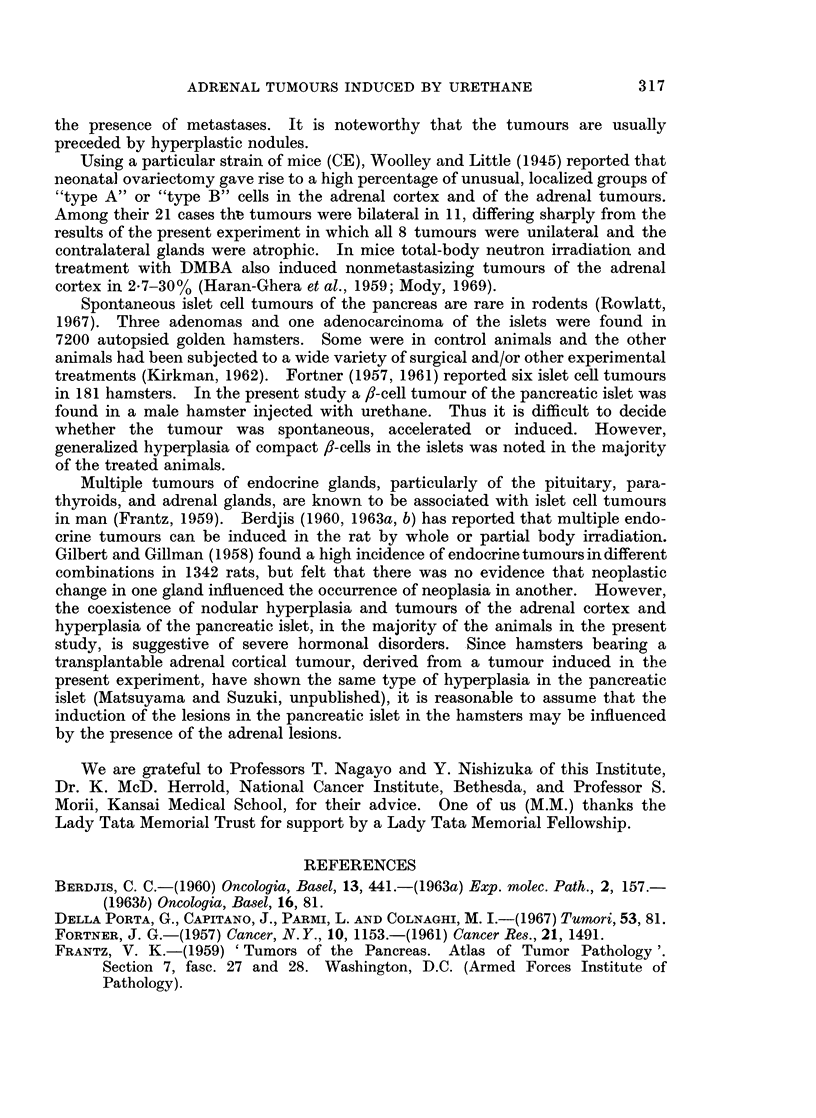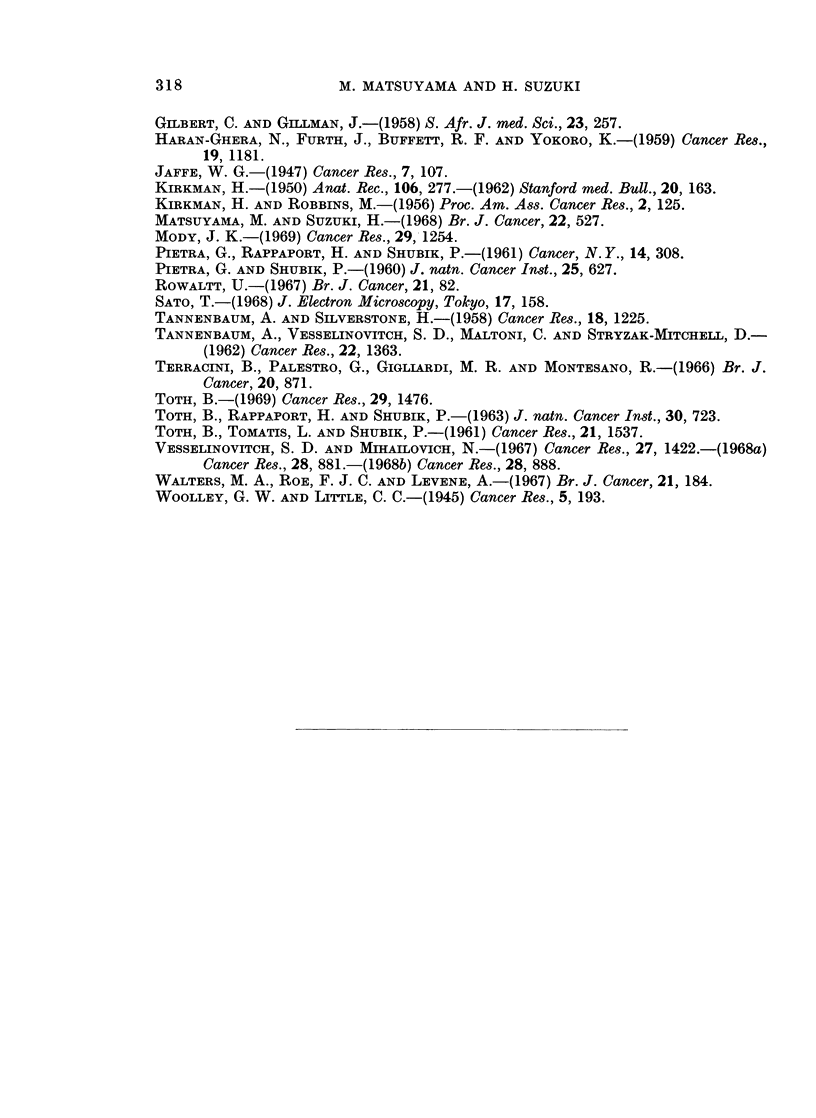# Adrenal tumours and endocrine lesions induced in Syrian hamsters by urethane injected during suckling period.

**DOI:** 10.1038/bjc.1970.36

**Published:** 1970-06

**Authors:** M. Matsuyama, H. Suzuki

## Abstract

**Images:**


### ADRENAL TUMOURS AND ENDOCRINE LESIONS INDUCED IN SYRIAN HAMSTERS BY URETHANE INJECTED DURING SUCKLING PERIOD

M. MATSUYAMA AND H. SUZUKI

*From the Aichi Cancer Center Research Institute, Nagoya, Japan*

Received for publication January 21, 1970

**SUMMARY.—Suckling Syrian golden hamsters were injected subcutaneously with 10% saline solution of urethane, at a dose of 1 mg./g. of body weight, once a week for 6 weeks. The majority of the animals, which survived more than 52 weeks, developed nodular hyperplasia of the adrenal cortex and of the pancreatic islet. Eight adrenal cortical tumours, including two metastasizing ones, and a β-cell tumour of the pancreatic islet were also found in the 28 treated hamsters. The histology of the adrenal lesions suggests that there are possible progressive steps from the hyperplastic nodules to the development of the tumours.**

URETHANE has been found to be a multipotential carcinogen in several strains of mice. Tumours of various organs including the lung, liver, ovary, thymus, lymph node, Harderian gland and others, are induced (Tannenbaum and Silverstone, 1958; Della Porta *et al.*, 1967; Vesselinovitch and Mihailovich, 1967; Matsuyama and Suzuki, 1968). Urethane also has a broad spectrum of action in rats inducing pulmonary adenomas, hepatomas, brain tumours, mammary fibroadenomas, and uterine sarcomas (Jaffe, 1947; Tannenbaum *et al.*, 1962; Vesselinovitch and Mihailovich, 1968*a* and *b*). The target organs for this carcinogen in Syrian hamsters differ from those in mice and rats. Melanotic tumours of the skin and papillomas of the forestomach are induced when this chemical is administered to hamsters in the drinking water (Pietra and Shubik, 1960; Toth, Tomatis and Shubik, 1961).

This report describes the effects of urethane given by subcutaneous injection to suckling Syrian golden hamsters.

### MATERIALS AND METHODS

*Experimental animals.*—Syrian golden hamsters, obtained from Dr. H. Uno, Department of Pathology, Nagoya University, Nagoya, and bred in our laboratory by sister-to-brother mating since 1965, were used. The colony originally came from the Cancer Research Institute, New England Deaconess Hospital, Boston, to the National Institute of Health, Tokyo, in 1959, and then to Dr. Uno in 1964.

*Experimental procedure.*—Animals from seven litters were given, in the interscapular region, 6-weekly subcutaneous injections of a 10% saline solution of urethane (E. Merck, Darmstadt) at a dose of 1 mg./g. of body weight. The injections were started at 7 days of age. Another ten litters that received no treatment served as controls. Following treatment the animals were kept separate from their mothers for about 3 hours to avoid maternal cannibalism. The litters were weaned and separated by sex at 6 weeks of age. They were housed in aluminium cages with sawdust bedding and were given CMF diet (Oriental Yeast, Tokyo) and water *ad libitum.*

The hamsters injected with urethane were permitted to live out their life span. Animals found in a semi-comatose condition were killed. The experiment was terminated at the end of 124 weeks, because the last female hamster injected with urethane died on the 728th day and the last male died on the 864th day. The surviving controls were killed at 104 weeks for females and 124 weeks for males. Complete necropsies were performed on all animals killed or found dead. The adrenals, thyroid gland, pituitary, ovaries, testes, pancreas, lungs, liver, spleen, Harderian glands, and other organs which showed grossly visible tumours were fixed in 10% formalin solution, sectioned and stained with haematoxylin and eosin. For electron microscopy, other areas of the pancreas were fixed in 3% glutaraldehyde buffered with 0·1 M phosphate for 3 hours and postfixed in 2% osmium tetroxide buffered with 0·1 M phosphate for 3 hours. The tissue blocks were dehydrated in graded ethanols, and then embedded in Epon 812. Ultrathin sections were cut on an LKB ultratome and stained with uranyl acetate and lead (Sato, 1968). Grids were studied with an Hitachi HU-11B electron microscope. Routine determinations of blood sugar were performed by using Dextrostix (Ames Japan, Tokyo).

### RESULTS

The survival, localization and frequency of tumours and lesions observed in the experimental and control groups are shown in Table I. The 6-weekly injections of urethane significantly reduced the survival. Most of the animals, which were injected with urethane and survived more than 52 weeks, became semicomatose for 2–6 days before death, and were severely emaciated. They usually showed severe hypoglycaemia with a blood glucose below 40 mg./ml. At autopsy a small to moderate volume of clear ascitic fluid was present and nodules of histiocytosis in atrophic spleens were noted in many of these animals.

*Adrenal hyperplasia.*—Most of the hamsters injected with urethane developed adrenal hyperplasia whereas this lesion was observed in only a few of the control animals (Table I). The lesion was first found in an injected, female hamster killed at 377 days after birth. In the control animals this was observed at 510 postnatal days. The surface of the adrenals was either translucent and normal appearing or characterized by the presence of whitish yellow pin-point spots. Histologically there were three types of nodular hyperplasia, including small spindle-shaped cells, densely packed medium-sized cells, or large eosinophilic granular cells. The nodular hyperplasia was bilateral and multicentric. Thus, the same adrenal frequently contained several nodules which varied in size and showed different cell types. These lesions seemed to originate usually from the zona glomerulosa and compressed the zona fasciculata and the zona reticularis (Fig. 1). Some of them showed marked displacement of the medulla (Fig. 2). In these cases a few mitotic figures were seen and a capsule bordering these lesions was lacking.

*Adrenal tumours.*—Macroscopical adrenal cortical tumours were produced in 8 hamsters (6 females and 2 males) injected with urethane (Table I). The first tumour was found in a female hamster killed at 492 days after birth. In the

TABLE I.—*Induction of Tumours and Other Lesions in Hamsters Injected with Urethane During Suckling Period*

| Treatment | Sex | Survival time (weeks) | Adrenal hyperplasia No. of animals | (%*) | Adrenal tumours No. of animals | (%*) | β-cell proliferation in pancreatic islet No. of animals | (%*) | Other lesions |
|---|---|---|---|---|---|---|---|---|---|
| Urethane† | F | 0 6 26 52 78 104<br>30 26 25 20 12 0 | 19 | (95) | 6 | (30) | 17 | (85) | 3 adenomatous hyperplasia of thyroid<br>19 spleen nodules of histiocytosis.<br>3 melanomas of skin and eyelids<br>4 papillomas of forestomach<br>3 haemangiomas of liver<br>1 adenocarcinoma of exocrine pancreas<br>2 cysts of liver |
| | M | 11 9 9 8 6 4 | 7 | (88) | 2 | (25) | 7 | (75) | 1 β-cell tumour of pancreatic islet<br>7 spleen nodules of histiocytosis<br>1 haemangioma of liver<br>2 cysts of liver |
| None | F | 31 27 26 25 21 12‡ | 9 | (36) | 0 | (—) | 2 | (8) | 2 cysts of liver<br>1 papilloma of forestomach |
| | M | 34 31 30 30 26 20‡ | 9 | (30) | 0 | (—) | 3 | (10) | 1 adenomatous hyperplasia of thyroid<br>3 cysts of liver |

* Percentages are given on survivors at 52 weeks after birth.

† Each animal was given 6-weekly subcutaneous injections at a dose of 1 mg./g. of body weight, starting at 7 days of age.

‡ These were killed 104th week for the females and in 124th week for the males, and the experiment was terminated.

males the tumour developed later (788 and 815 days after birth). Two of the tumours were yellow, soft and bean-size (Fig. 3). Histologically narrow rims of nontumourous cortical tissue, which contained hyperplastic nodules of different types, surrounded the tumours. The border of the tumours was well defined, but no capsule was evident. The tumours consisted of medium sized, densely packed cells with eosinophilic cytoplasm, showing a trabecular pattern (Fig. 4). A few mitotic cells were noted. Four others were yellow-white, soft, and finger-tip sized tumours. No cortical tissue was observed around the tumour masses, except cysts. The tumours consisted of two or three types of cells; small spindle-shaped cells, medium sized densely packed cells, and/or large granular cells, with intermingled areas of these cell types (Fig. 5 and 6). Mitotic cells were not frequent. There was no infiltration or metastases in other organs. The tumours were unilateral, and the contralateral glands were slightly atrophic, weighing 5–10 mg. The remaining two tumours in the female hamsters were of thumb size and had infiltrations and/or metastases in the kidney, liver, periportal- and perigastric lymph nodes, and lungs (Fig. 7). The tumours consisted of large polygonal and pleomorphic cells (Fig. 8), containing large areas of coagulation necrosis and haemorrhage.

*Other endocrine lesions.*—The majority of hamsters injected with urethane showed proliferation of densely packed cells in the centre of the pancreatic islets (Table I and Fig. 9). This $\beta$-cell proliferation was first noticed in a female hamster killed at 425 days after birth. In the control animals this was found only in the hamsters killed at the end of the experiment, 104 weeks after birth for the females and 124 weeks for the males. These proliferating cells were oval with vesicular nuclei and narrow, eosinophilic-granular cytoplasm, in which a few but definite $\beta$-cell granules were found submicroscopically (Fig. 10). A $\beta$-cell tumour was found in a male hamster injected with urethane, age 788 days (Table I). The tumour, which was in the deeper area of the pancreas, was noticed by red colour of a blood lake contained in the mass. It was ovoid, of rice size, and surrounded by a thick capsule. The tumour cells were large and stained slightly eosinophilic, and had an intimate relationship to abundant capillaries (Fig. 11). A number of dark stained and pyknotic cells was scattered among the clear large cells, but mitoses were seldom identified. Submicroscopically it was diagnosed as a $\beta$-cell tumour of the pancreatic islet.

Adenomatous hyperplasia of the thyroid gland was also found in three female hamsters injected with urethane and in one male control animal (Table I and Fig. 12). The pituitaries, ovaries, and testes were severely atrophic in the animals injected with urethane. In addition to the endocrine lesions, other types of lesions were also found in a few treated and control hamsters (Table I).

### DISCUSSION

Neonatal injection of 150 $\mu$g. of urethane failed to induce tumours in Syrian golden hamsters (Walters, Roe and Levene, 1967). This was probably because the dose, which may correspond to 0·06–0·07 mg./g. of body weight, was too low. The administration of urethane in the drinking water (0·2%) to 8- to 10-week-old hamsters gave rise to melanotic tumours of the skin in a moderate percentage (Pietra and Shubik, 1960). Using larger doses (0·2 and 0·4%) and younger animals, 5- to 7-weeks old, Toth, Tomatis and Shubik (1961) induced melanotic tumours in a higher percentage. Papillomas and carcinomas of the forestomach, malignant lymphomas, and liver tumours also developed. The present experiment, using suckling hamsters and subcutaneous injections of still larger doses, succeeded in producing endocrine lesions besides melanotic tumours. This may be due to the age of the animals used. Such a change in the target organs of a carcinogen because of difference in age of animals has been demonstrated in the types of mouse tumours induced by 7,12-dimethylbenz(a)anthracene (DMBA), dimethylnitrosamine, and urethane (Pietra, Rappaport, and Shubik, 1961; Toth Rappaport, and Shubik, 1963; Terracini *et al.*, 1966; Della Porta *et al.*, 1967; Vesselinovitch and Mihailovich, 1967).

Spontaneous adrenal cortical tumours in hamsters are not infrequent and 4 adenomas were found in 51 untreated animals (Kirkman, 1950). However, adrenal tumours in hamsters induced by carcinogens have rarely been reported in the literature. Kirkman and Robbins (1956) found 35 adrenal cortical tumours, one a carcinoma, among 64 male and female hamsters that lived with subcutaneously implanted testosterone pellets for 608–950 days. Toth (1969) recently reported that 10 adrenal tumours were found in 60 young adult hamsters injected intravenously with 3 mg. of DMBA. In the present experiment 8 adrenal tumours, 2 with metastases, were found in 28 hamsters injected with urethane. Multiple areas of nodular hyperplasia were also present in the adrenal cortex. These two types of adrenal lesions had three different histological patterns; small spindle cell type, medium sized compact cell type and large granular cell type. It was therefore difficult to establish specific criteria for the histological diagnosis of hyperplasia, adenoma and carcinoma. Thus a simple biological classification was adopted, determined by the gross appearance of tumours and

---

EXPLANATION OF PLATES

FIG. 1.—Subcapsular nodular hyperplasia of granular cell type compresses the zona fasciculata. H. and E. × 59.

FIG. 2.—Nodular hyperplasia of compact cell type replaces the zona fasciculata and the zona reticularis, and invades into the medulla. H. and E. × 59.

FIG. 3.—Bean sized tumour in the left adrenal gland, with area of subcapsular bleeding. Two-thirds was removed for transplantation and for electron microscopy. Atrophy of right adrenal (lower). × 2·4.

FIG. 4.—Same hamster shown in Fig. 3. Tumour of medium sized compact cell type, showing trabecular pattern. H. and E. × 445.

FIG. 5.—Area of adrenal tumour consisting of spindle cells. H. and E. × 445.

FIG. 6.—Same hamster shown in Fig. 5. Another area of the tumour consisting of large granular cells, showing large discrete nucleoli. H. and E. × 445.

FIG. 7.—Tumour in the right adrenal gland with the infiltration into the right kidney and liver. Multiple metastatic nodules in the lungs (upper) and periportal- and perigastric-lymph nodes (lower right) are also shown. × 1·1.

FIG. 8.—Same hamster shown in Fig. 7. Tumour cells are large and pleomorphic, with many mitotic figures. H. and E. × 445.

FIG. 9.—Same hamster shown in Fig. 7 and 8. In the centre of the islet proliferation of small, eosinophilic stained cells are shown. H. and E. × 289.

FIG. 10.—Electron micrograph taken from a pancreatic islet of a female hamster injected with urethane. A β-cell with narrow cytoplasm containing a few β-cell granules (arrows) is shown. Stained with uranyl acetate and lead. × 7320.

FIG. 11.—Same hamster shown in Fig. 3 and 4. Beta-cell tumour of the pancreatic islet consisting of cells which have large vesicular nuclei and irregular shaped, slightly eosinophilic cytoplasm, showing mosaic pattern and intimate relationship with capillaries. H. and E. × 445.

FIG. 12.—Same hamster shown in Fig. 7, 8, and 9. Adenomatous hyperplasia of the thyroid. H. and E. × 67.

Matsuyama and Suzuki.

Matsuyama and Suzuki.

9

10

11

12

Matsuyama and Suzuki.

the presence of metastases. It is noteworthy that the tumours are usually preceded by hyperplastic nodules.

Using a particular strain of mice (CE), Woolley and Little (1945) reported that neonatal ovariectomy gave rise to a high percentage of unusual, localized groups of "type A" or "type B" cells in the adrenal cortex and of the adrenal tumours. Among their 21 cases the tumours were bilateral in 11, differing sharply from the results of the present experiment in which all 8 tumours were unilateral and the contralateral glands were atrophic. In mice total-body neutron irradiation and treatment with DMBA also induced nonmetastasizing tumours of the adrenal cortex in 2·7–30% (Haran-Ghera *et al.*, 1959; Mody, 1969).

Spontaneous islet cell tumours of the pancreas are rare in rodents (Rowlatt, 1967). Three adenomas and one adenocarcinoma of the islets were found in 7200 autopsied golden hamsters. Some were in control animals and the other animals had been subjected to a wide variety of surgical and/or other experimental treatments (Kirkman, 1962). Fortner (1957, 1961) reported six islet cell tumours in 181 hamsters. In the present study a $\beta$-cell tumour of the pancreatic islet was found in a male hamster injected with urethane. Thus it is difficult to decide whether the tumour was spontaneous, accelerated or induced. However, generalized hyperplasia of compact $\beta$-cells in the islets was noted in the majority of the treated animals.

Multiple tumours of endocrine glands, particularly of the pituitary, parathyroids, and adrenal glands, are known to be associated with islet cell tumours in man (Frantz, 1959). Berdjis (1960, 1963*a*, *b*) has reported that multiple endocrine tumours can be induced in the rat by whole or partial body irradiation. Gilbert and Gillman (1958) found a high incidence of endocrine tumours in different combinations in 1342 rats, but felt that there was no evidence that neoplastic change in one gland influenced the occurrence of neoplasia in another. However, the coexistence of nodular hyperplasia and tumours of the adrenal cortex and hyperplasia of the pancreatic islet, in the majority of the animals in the present study, is suggestive of severe hormonal disorders. Since hamsters bearing a transplantable adrenal cortical tumour, derived from a tumour induced in the present experiment, have shown the same type of hyperplasia in the pancreatic islet (Matsuyama and Suzuki, unpublished), it is reasonable to assume that the induction of the lesions in the pancreatic islet in the hamsters may be influenced by the presence of the adrenal lesions.

We are grateful to Professors T. Nagayo and Y. Nishizuka of this Institute, Dr. K. McD. Herrold, National Cancer Institute, Bethesda, and Professor S. Morii, Kansai Medical School, for their advice. One of us (M.M.) thanks the Lady Tata Memorial Trust for support by a Lady Tata Memorial Fellowship.

### REFERENCES

BERDJIS, C. C.—(1960) *Oncologia, Basel*, **13**, 441.—(1963*a*) *Exp. molec. Path.*, **2**, 157.—(1963*b*) *Oncologia, Basel*, **16**, 81.

DELLA PORTA, G., CAPITANO, J., PARMI, L. AND COLNAGHI, M. I.—(1967) *Tumori*, **53**, 81.

FORTNER, J. G.—(1957) *Cancer, N.Y.*, **10**, 1153.—(1961) *Cancer Res.*, **21**, 1491.

FRANTZ, V. K.—(1959) 'Tumors of the Pancreas. Atlas of Tumor Pathology'. Section 7, fasc. 27 and 28. Washington, D.C. (Armed Forces Institute of Pathology).

GILBERT, C. AND GILLMAN, J.—(1958) *S. Afr. J. med. Sci.*, **23**, 257.
HARAN-GHERA, N., FURTH, J., BUFFETT, R. F. AND YOKORO, K.—(1959) *Cancer Res.*, **19**, 1181.
JAFFE, W. G.—(1947) *Cancer Res.*, **7**, 107.
KIRKMAN, H.—(1950) *Anat. Rec.*, **106**, 277.—(1962) *Stanford med. Bull.*, **20**, 163.
KIRKMAN, H. AND ROBBINS, M.—(1956) *Proc. Am. Ass. Cancer Res.*, **2**, 125.
MATSUYAMA, M. AND SUZUKI, H.—(1968) *Br. J. Cancer*, **22**, 527.
MODY, J. K.—(1969) *Cancer Res.*, **29**, 1254.
PIETRA, G., RAPPAPORT, H. AND SHUBIK, P.—(1961) *Cancer, N.Y.*, **14**, 308.
PIETRA, G. AND SHUBIK, P.—(1960) *J. natn. Cancer Inst.*, **25**, 627.
ROWALTT, U.—(1967) *Br. J. Cancer*, **21**, 82.
SATO, T.—(1968) *J. Electron Microscopy, Tokyo*, **17**, 158.
TANNENBAUM, A. AND SILVERSTONE, H.—(1958) *Cancer Res.*, **18**, 1225.
TANNENBAUM, A., VESSELINOVITCH, S. D., MALTONI, C. AND STRYZAK-MITCHELL, D.—(1962) *Cancer Res.*, **22**, 1363.
TERRACINI, B., PALESTRO, G., GIGLIARDI, M. R. AND MONTESANO, R.—(1966) *Br. J. Cancer*, **20**, 871.
TOTH, B.—(1969) *Cancer Res.*, **29**, 1476.
TOTH, B., RAPPAPORT, H. AND SHUBIK, P.—(1963) *J. natn. Cancer Inst.*, **30**, 723.
TOTH, B., TOMATIS, L. AND SHUBIK, P.—(1961) *Cancer Res.*, **21**, 1537.
VESSELINOVITCH, S. D. AND MIHAILOVICH, N.—(1967) *Cancer Res.*, **27**, 1422.—(1968*a*) *Cancer Res.*, **28**, 881.—(1968*b*) *Cancer Res.*, **28**, 888.
WALTERS, M. A., ROE, F. J. C. AND LEVENE, A.—(1967) *Br. J. Cancer*, **21**, 184.
WOOLLEY, G. W. AND LITTLE, C. C.—(1945) *Cancer Res.*, **5**, 193.